# Supplementary material for: Patterns of Intron Gain and Loss in Fungi
Source: PLoS Biol. 2004 Nov 30;2(12):e422. doi: 10.1371/journal.pbio.0020422 (PMC532390; doi:10.1371/journal.pbio.0020422)
Supplement: Table S1 — Also available at http://genes.mit.edu/NielsenEtAl/. (4.3 MB ZIP). [file pbio.0020422.st001.zip › NielsenEtAl/html/1213.html]

AN5789.1.NCU00777.1.MG08705.1.FG05728.1


```
 CLUSTAL W (1.82) Multiple Sequence Alignments - Introns Inserted


Sequence 1: NCU00777.1	278 aa
Sequence 2: FG05728.1	280 aa
Sequence 3: MG08705.1	279 aa
Sequence 4: AN5789.1	279 aa
Alignment Length: 282 aa
Number Identitical Residues: 184 aa
Alignment Score (without introns) 7895


MG08705.1 	MSRPEDTL2AADVHYNDTEARKYTTSSRIQNIQAAMTRRALELLELTSPSLILDVGCGSG
NCU00777.1	MSRPEDTL2AADVHYDDTEARKYTTSSRIQNIQASMTRRALELLDLKSPSFILDIGCGSG
FG05728.1 	MSRPEDTL2AADVHYDDTEARKYTTSSRIQNIQASMTRRALELLDLKSPSLILDIGCGSG
AN5789.1  	MSRPEDIL2PPDLFYDDNESRKYTTSSRIRNIQSDMTHRALELLDLKAPSLILDVGCGSG
          	****** * ..*:.*:*.*:*********:***: **:******:*.:**:***:*****

MG08705.1 	LSGEILTAEDPADGGPHTWIGMDVSPSMLDVALQRDVAGDLMLADIGQGVPFRAGSFDAA
NCU00777.1	LSGEILSDVPEDEGGPHVWVGMDISPSMLDTALQRDVEGDLFLADIGQGVPFRAGSFDAA
FG05728.1 	LSGEILSSVEPEEGGPHTWIGMDVSPSMLDIALQRDVEGDLMLADIGQGVPFRAGTFDAA
AN5789.1  	LSGEILSQEPREQGGPHTWIGMDISPSMLDIALQRGVEGDLFLADIGQGVPFRPGTFDAA
          	******:     :****.*:***:****** ****.* ***:***********.*:****

MG08705.1 	ISISAIQWLCNAESSDTSPLGRLSRFFNGLYASLRRGGRAVCQFYPKNDTQRDMIAGAAK
NCU00777.1	ISISAIQWLCNAETSDTAPSTRLSRFFNGLYASLKRGGRAVCQFYPKNDVQKQMITAAAV
FG05728.1 	ISISAIQWLCSAETSDTSPVGRLTRFFNGLYASLKRGGRAVCQFYPKNDDQRNMITQAAV
AN5789.1  	ISISAIQWLCNAETSDVSPEGRLRRFFEGLFASLRRGGRAVCQFYPKNDAQRTMISGAAI
          	**********.**:**.:*  ** ***:**:***:************** *: **: ** 

MG08705.1 	KAGFAAGLLEDDPDTKNKKVYLVLTVGGSAHDGSGGDITDVVKGLDNVDVLDVSRKP--K
NCU00777.1	KAGFGAGLLEDDPNTKNVKLYLVLTVGKDATG--GKDISTVVENMDGVDILDQRKKG--K
FG05728.1 	KAGFGAGMLEDDPGTKNQKLYLVLTVGGGDLQKKGGDITGVVDGMEGVDVEDARRSI--K
AN5789.1  	KAGFGAGILEDDPGTKNSKLYLVLTVGGGGLQ---GDITGVVDGMNDVDIMDARRKAQEH
          	****.**:*****.*** *:******* .       **: **..::.**: *  :. ..:

MG08705.1 	AGR-DMRKGGKAWIVKKKEQMERKGKVVKASSKYTGRKRKPAF
NCU00777.1	GGKGEIKKGSKGWIIKKKEQMERKGKIVKATSKYTGRKRRIQF
FG05728.1 	THAPNMSKGSKQWIVKKKEQMERKGKIVKATSKYTGRKRRIQF
AN5789.1  	GKATISRKGDKAWILRKKEQMAKKGKVVKANSKYTGRKRRPAF
          	       **.* **::***** :***:***.********:  *
```
